# Prediction of Multisite Pain Incidence in Adolescence Using a Machine Learning Approach: A 2‐Year Longitudinal Study

**DOI:** 10.1002/hsr2.70252

**Published:** 2024-12-10

**Authors:** Laura Joensuu, Ilkka Rautiainen, Arto J. Hautala, Kirsti Siekkinen, Katariina Pirnes, Tuija H. Tammelin

**Affiliations:** ^1^ Faculty of Sport and Health Sciences University of Jyväskylä Jyväskylä Finland; ^2^ Likes Jamk University of Applied Sciences Jyväskylä Finland

**Keywords:** child health, epidemiology, musculoskeletal health, predictive modeling

## Abstract

**Background and Aims:**

Multisite pain is a prevalent and significant issue among adolescents, often associated with adverse physical, psychological, and social outcomes. We aimed to (1) predict multisite pain incidence in the whole body and in the musculoskeletal sites in adolescents, and (2) explore the sex‐specific predictors of multisite pain incidence using a novel machine learning (ML) approach (random forest, AdaBoost, and support vector classifier).

**Methods:**

A 2‐year longitudinal observational study (2013–2015) was conducted in a population‐based sample of Finnish adolescents (*N* = 410, 57% girls, 12.5 years (SD = 1.2) at baseline). Three different data sets were used. First data included 48 pre‐selected variables relevant for adolescents' health and wellbeing. The second data included nine physical fitness variables related to the Finnish national ‘Move!’ monitoring system for health‐related fitness. The third data set included all available baseline data (392 variables). Multisite pain was self‐reported weekly pain during the past 3 months manifesting in at least three sites and not related to any known disease or injury. Musculoskeletal pain sites included the neck/shoulder, upper extremities, chest, upper back, low back, buttocks, and lower extremities. Whole body pain sites also included the head and abdominal areas.

**Results:**

Overall, 16% of boys and 28% of girls developed multisite pain in the whole body and 10% and 15% in the musculoskeletal area during the 2‐year follow‐up. The prediction ability of ML reached area under the receiver operating characteristic curve 0.78 at highest but remained mainly < 0.7 for the majority of the methods. With ML, a broad variety of predictors were identified, with up to 33 variables showing predictive power in girls and 13 in boys.

**Conclusion:**

The results highlight that rather than any isolated variable, a variety of factors contribute to future multisite pain.

## 1 Introduction

Pain is common in adolescents [1]. Long‐lasting pain in at least two bodily locations is reported by at least every tenth and up to every third adolescent in large cohort studies [2], with musculoskeletal locations most common sites for pain [3]. This co‐occurrence of pain is typical in the adolescent population [4], and hence, multisite pain is recommended to be considered in clinical practice over isolated pain sites [2]. While the adolescent population is relatively free from many disabling health outcomes, experiences of pain are associated with a lesser ability to conduct daily activities. This association follows a dose‐response pattern where more pain sites are associated with a higher degree of disability [3]. The relevance of multisite pain in adolescence is stressed by pediatric pain researchers [5]. Pain in adolescence is associated with a broad range of adverse outcomes, from limitations in school attendance and hobbies to reduced quality of life and depressive symptoms, with especially multisite pain affecting several areas of daily living [3, 6, 7, 8]. Alarmingly, experiences of multisite pain are common in adolescents [3]. Furthermore, pain experiences tend to track from childhood and adolescence into adulthood [9, 10], with high relevance to, e.g. future work disability [11].

Previous studies have found several cross‐sectional correlates with pain. Various biological, psychosocial, and lifestyle factors, such as age, pubertal status, overweight or obesity, symptoms of anxiety and depression, chronic health problems, frequent change of residence, poor academic achievement, leisure screen time, fewer interactions with peers, unhealthy lifestyles (e.g., sedentary behavior, screen time, inadequate sleep and smoking), and excessive physical activity, especially in a technical, team, strength, or extreme sports increase the odds for overall or musculoskeletal pain [1, 2, 12, 13, 14, 15]. Of these, specifically sedentary behavior, overweight/obesity, and smoking reportedly correlate with multisite pain especially in adolescent girls [12]. Previous findings have also shown that psychological distress and anxiety associate with multisite musculoskeletal pain regardless of the gender [8]. In addition, physical fitness is suggested to be associated with pain in adolescents. Especially the associations of flexibility and muscular fitness with musculoskeletal pain have been of great interest. Findings remain persistently inconclusive, although the majority of studies have focused on examining a specific pain site [16, 17]. Despite the inconclusive evidence, proper functioning of the musculoskeletal system, i.e adequate exertion of force, fatigue resistance, and range of motion in the body, is a rationale for many health‐related large‐scale monitoring and surveillance systems to implement fitness testing at the population level [16, 18]. Previous findings also indicate that girls report pain more often than boys [2, 3], and the correlates of pain might be sex‐specific, potentially due to differences in maturation, pain tolerance, or coping behaviors between sexes [2, 12].

Less is known about the predictors of pain incidence. Predicting the future onset of multisite pain has proven to be challenging despite the broad range of potential explanatory factors. For example, Paananen et al. [13] did not find statistically significant predictors for multisite musculoskeletal pain incidence in a 2‐year follow‐up study. Recently, machine learning (ML)‐based pattern recognition approaches have emerged as promising alternatives to traditional statistical approaches in modeling complex phenomenon in various areas of society, including health care [19]. In this approach, a hypothesis‐free data‐driven methods are used to handle complex, high‐dimensional data for predictive tasks [20]. Therefore, the aim of this study was to (1) predict multisite pain incidence in the musculoskeletal and whole body sites in adolescents and (2) explore the sex‐specific determinants of multisite pain incidence utilizing a novel ML approach.

## 2 Methods

### 2.1 Study Population

**Figure 1:**
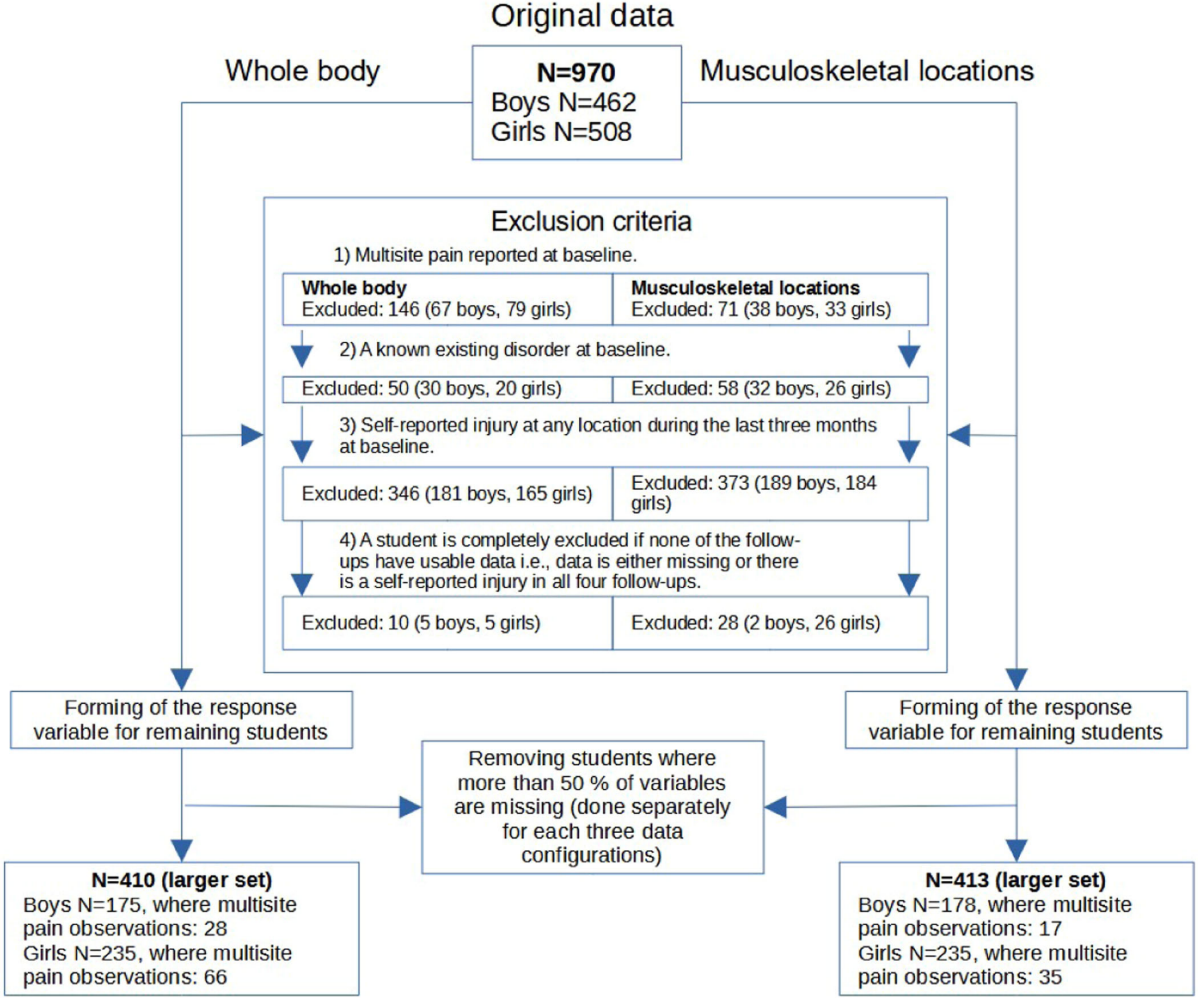
Flow chart of the exclusion process.

This study was part of a research entity related to the Finnish Schools' on the Move program focusing on physical activity and wellbeing among children and adolescents [21]. A longitudinal observational study was conducted between January 2013 and June 2015. A total of 1778 students from nine Finnish schools were invited to participate. Out of these, 970 students (53% girls) provided signed written consent with their main caregiver and participated in the study. After excluding students with possible confounding factors at baseline (such as guardian‐reported existing chronic illnesses or disorders, injuries, existing multisite pain, and more than 50% of missing data), the final sample consisted of 410 apparently healthy participants (57% girls) (Figure 1).

The study setting and measurements were approved by the Ethics Committee of the University of Jyväskylä, and all procedures were conducted in accordance with the principles outlined in the Declaration of Helsinki. Participants had the option to discontinue their involvement at any point during the research. All measurements were conducted by trained personnel.

### 2.2 Outcome

Pain incidence was defined as the new onset of multisite pain at any time point during the 2‐year follow‐up. Pain symptoms were screened four times after baseline with structured questionnaires at 6‐month intervals: “How often you have had symptoms in the last 3 months (in body parts A–I in the pictures below)? Mark the appropriate option. Headache (A), Neck and shoulder pain/ache (B), Upper extremities pain/ache (C), Chest pain/ache (D), Upper back pain/ache (E), Low back pain/ache (F), Stomach ache (G), Buttocks pain/ache (H), Lower extremities pain o/ache (I)” [22]. The question was supported by an illustration of the described body parts. The answering options were: “Almost daily, More than once a week, About once a week, About once a month, Seldom or never.” The used approach combines the information regarding pain symptom, frequency and location. Students also reported if the pain originated from trauma:”Have you injured any of the above‐mentioned and pictured pain areas during the previous 3 months (e.g., fallen, stumbled, being hurt during sport, etc.)?” The answer options were “yes” or “no” and provided additional information related to the injured body area.

Multisite pain was defined as reported weekly pain (almost daily, more than once a week, or about once a week) in at least three sites during the past 3 months. Pains due to traumatic causes were excluded from the analysis. In this study, multisite pain was reported separately for the whole body and musculoskeletal locations. The pain reported in at least three sites was selected to reflect the disabling form of multisite pain. Previously, multisite pain measured in a similar manner has shown increased prevalence of difficulties in falling asleep, sitting during school lesson, disturbances during 1 km walking, disturbances during exercise class and difficulties in participating to leisure time activities in adolescents [3]. Musculoskeletal pain sites included the neck/shoulder, upper extremities, chest, upper back, low back, buttocks, and lower extremities. Whole body pain sites included additionally head and abdominal areas. The categorization was based on previous literature [3].

### 2.3 Predictive Variables

We aimed to determine the predictors of multisite pain incidence using three different data sets. First, we included 48 selected baseline variables relevant to adolescents' physical activity, fitness, health, and wellbeing [23]. The data included information on participants' basic demographics, physical, psychosocial, and lifestyle characteristics and is presented in detail elsewhere [23]. The results of these analyses are presented in the main text.

Secondly, we used baseline physical fitness measurements belonging to the Finnish national ‘Move!’ monitoring and surveillance system for health‐related fitness [24] (nine baseline variables include 20‐m shuttle run, push‐up, curl‐up, 5‐leaps test, throwing–catching combination test, squat, lower‐back extension in sitting posture, and shoulder stretch (measurement protocols described in detail elsewhere) [25]. Annually, approximately 100,000 children and adolescents (approximately 96% of the relevant age groups) participate in ‘Move!’ creating a unique database for health‐enhancing policies [26].

Thirdly, a data‐driven approach was used with the whole available data (392 baseline variables) to explore potential novel predictors of multisite pain incidence. Data included extensive information on students' self‐reported, and device‐based demographics, physical and psychosocial characteristics, and physical activity.

### 2.4 Analytical Procedures

The random forest (RF), AdaBoost, and support vector classifier (SVC) methods were applied. The RF method was the default, and latter two methods were utilized only to confirm the prediction ability of the RF method. With AdaBoost and SVC we used only the 48 selected baseline variables for analyses. All analyses were performed using MATLAB R2023b with the Statistics and Machine Learning Toolbox and conducted separately for both sexes. Initial preprocessing and creation of the outcome variable were made using the Python programming language.

Firstly, RF is an ML method where multiple de‐correlated decision trees are grown to form a forest. Afterward, this forest is employed as a voting ensemble, where each tree provides an answer for the prediction task. The final prediction of the forest is the class that gets the most votes from the individual trees [27, 28]. Secondly, AdaBoost (adaptive boosting) adds multiple weak learners, like decision trees, to create a strong classifier. It sequentially trains each learner, focusing on instances misclassified by previous ones, thereby improving the overall model [29]. Finally, SVC aims to create a separating hyperplane that best separates the classes, maximizing the margin between them [28].

Ten‐fold cross‐validation (CV) was employed for model assessment. During CV, the data for each prediction task was divided into 10 subsamples called folds. Nine of these folds, 90% of the whole data set, were used as the training data to fit the RF, AdaBoost or SVC model, while onefold, 10% of the data, was used as the validation data. This procedure was repeated 10 times in a rotating manner, where eventually all the folds had been employed for training and validation. Thus, all the presented results are based on 10 separate data‐driven prediction models.

For each of the 10 CV folds in RF, the trained model was employed to predict the out‐of‐bag (OOB) observations i.e., those observations which were not utilized during the training of each tree, and the validation portion of the data. The main metric recorded was the area the under receiver operating characteristic curve (AUC). T‐tests were performed in MATLAB for the OOB and validation data AUC results to determine if the means of the CV folds were significantly (*p* < 0.05) above the random level of 0.5. Further analyses regarding the predictive power of each variable were conducted only in those cases where AUC 95% confidence interval (CI) did not violate the 0.5 threshold. A similar setup was used for AdaBoost and SVC. However, since OOB observations are exclusive to RF, they could not be utilized for performance estimation in these two methods.

RF requires choosing several hyperparameters i.e., options that define the model creation. F‐measure for the training data OOB observations was used as a target during Bayesian optimization [30], where several hyperparameters of the RF model were chosen in an automated fashion. For the AdaBoost and SVC models, hyperparameters were also tuned using Bayesian optimization, employing a nested fivefold CV for each fold. Please see Supporting Information S1: Supplementary methods for further information on the target measure, the hyperparameters, and other details concerning the RF, AdaBoost and SVC models.

The contribution of each variable to prediction was estimated using the RF's OOB observations by a permutation importance measure. A baseline result for the model in each CV fold was the accuracy of the model with the original data. To estimate the contribution of each variable, the values of the variables were permuted randomly. The procedure was repeated for all the variables separately, and the accuracy of the model with permutations was recorded for each variable. The accuracy obtained using the permuted variable was then subtracted from the baseline accuracy. The final permutation importance estimate for each variable was the mean of accuracy change for the 10 CV folds. T‐tests were employed also for the importance estimates. If the change was significantly (*p* < 0.05) over zero, the variable was seen as having predictive power. Furthermore, if the mean change was near zero or negative, the variable did not have importance in the prediction. MATLAB's predict function in the TreeBagger class was utilized to calculate the OOB predictions on the trained model. This function computed the weighted average of the class posterior probabilities over the trees. To provide an alternative perspective on the importance of individual predictors, SHAP (SHapley Additive exPlanations) values were extracted for the AdaBoost and SVC models. The results and details of the SHAP are presented in Supporting Information S1: Figures S4–11.

In further sensitivity analyses the class imbalance was considered, meaning that there are considerably less observations in diffuse idiopathic pain class, is a challenge in all explored settings. This issue was approached during the modeling in two separate ways. Firstly, in the RF, AdaBoost and SVC models by changing the default cost matrix of misclassification. Cost of misclassifying true pain class observations to no pain class (false negative classification) was increased to 2, while the other misclassification (false positive) was left to its default value 1.

Furthermore, as an alternate view, a synthetic minority oversampling technique for nominal and continuous data SMOTE‐NC [31], was utilized with RF to see if artificially balancing the training data by oversampling the pain class observations provided any performance improvements. Since SMOTE‐NC, available in Themis library in R, expected that there are no missing values in data, missForest imputation for mixed‐type data was utilized before oversampling. As a limitation, due to artificially manipulating the training data, the OOB observations could not be meaningfully utilized during this experiment and only non‐manipulated validation data for each CV fold was used when estimating the performance measures.

When estimating the importance of individual variables, the associated risk for each variable was examined with simple ROC analysis while acknowledging how the variables were coded in the data. The analysis was done separately from the RF model for the whole age‐adjusted data once without utilizing CV. The analysis was performed only for continuous and ordinal variables. The identified risk variables are presented in permutation importance estimate figures with a red panel.

## 3 Results

Descriptive information on the study sample is provided in Table 1. At baseline, headache (22.5%, 30.4%) and neck and shoulder pain (13.5%, 18.5%) were the most prevalent pain symptoms among boys and girls, respectively (Table 1).

**Table 1:** Selected subject demographics at baseline by sex.

*N* = 410	Boys	Girls
Characteristic		
*N*	175 (42.6%)	235 (57.3%)
Age (years)	12.5 (1.2)	12.5 (1.2)
Height (cm)	156.7 (10.9)	155.1 (9.6)
Weight (kg)	46.1 (12.2)	45.4 (10.1)
BMI (kg/m^2^)	18.5 (3.2)	18.7 (3.0)
Pain at different body sites		
Any pain (one or two sites)	59 (33.7%)	99 (42.1%)
Musculoskeletal pain sites		
Neck/shoulder (%)	23 (13.6%)	42 (18.5%)
Upper extremities (%)	7 (4.1%)	10 (4.4%)
Chest (%)	1 (0.6%)	2 (0.9%)
Upper back (%)	5 (3.0%)	5 (2.2%)
Low back (%)	8 (4.7%)	8 (3.5%)
Buttocks (%)	2 (1.2%)	6 (2.6%)
Lower extremities (%)	9 (5.3%)	13 (5.7%)
Other pain sites		
Head (%)	38 (22.5%)	69 (30.4%)
Abdominal (%)	18 (10.7%)	36 (15.9%)
Physical activity and fitness		
Moderate‐to‐vigorous physical activity (min/day)	58.3 (24.2)	47.4 (18.3)
Sedentary time (hours/day)	8.1 (1.3)	8.6 (1.1)
Physical fitness index (move‐index)	16.6 (4.1)	17.2 (3.6)

*Note:* Values are means and standard deviations for continues variables and proportions of participants for others.

Abbreviations: BMI, body mass index; move‐index, weighted sum of the Finnish national Move! monitoring system's fitness items.

### 3.1 Whole Body Multisite Pain Incidence

Sixteen percent of boys and 28.1% of girls experienced multisite pain incidents in the whole body area during the 2‐year follow‐up (Table 2). The ability of the ML approach to predict whole body multisite pain incidence reached an AUC 0.54 (95% CI: 0.51–0.57) for boys and 0.65 (0.64–0.67) for girls with the first data set and our default RF OOB observations (Table 2). Incorporating additional ML‐methods (AdaBoost, SVC) did not provide systematically or considerably better prediction ability (Table 2).

**Table 2:** Prediction ability of machine learning for multisite pain incidence among adolescents.

			Prediction ability				
Multisite pain incidence	Cases/N	Method	AUC (95% CI)	Sensitivity	Specificity	F‐measure	Balanced accuracy
All body sites							
Boys	28/175	RF	0.62 (0.51–0.73)	0.67 (0.51–0.83)	0.45 (0.28–0.63)	0.30 (0.23–0.37)	0.56 (0.49–0.63)
		RF (OOB)	0.54 (0.51–0.57)	0.64 (0.50–0.78)	0.54 (0.37–0.71)	0.32 (0.30–0.34)	0.59 (0.57–0.61)
		AdaBoost	0.47 (0.33–0.61)	0.17 (0.06–0.28)	0.74 (0.64–0.85)	0.24 (0.19–0.30)	0.46 (0.40–0.51)
		SVC	0.65 (0.44–0.86)	0.17 (0.06–0.28)	0.88 (0.82–0.94)	0.34 (0.29–0.40)	0.52 (0.47–0.58)
Girls	66/235	RF	0.67 (0.61–0.73)	0.59 (0.42–0.75)	0.62 (0.48–0.76)	0.44 (0.35–0.54)	0.60 (0.55–0.66)
		RF (OOB)	0.65 (0.64–0.67)	0.66 (0.60–0.72)	0.64 (0.56–0.71)	0.51 (0.50–0.53)	0.65 (0.64–0.67)
		AdaBoost	0.60 (0.50–0.70)	0.49 (0.33–0.65)	0.62 (0.47–0.76)	0.41 (0.34–0.49)	0.55 (0.48–0.63)
		SVC	0.63 (0.50–0.76)	0.65 (0.51–0.78)	0.47 (0.41–0.53)	0.42 (0.35–0.49)	0.56 (0.50–0.62)
Musculoskeletal sites							
Boys	17/178	RF	0.67 (0.54–0.81)	0.70 (0.48–0.92)	0.61 (0.50–0.72)	0.29 (0.23–0.36)	0.65 (0.57–0.73)
		RF (OOB)	0.64 (0.62–0.66)	0.69 (0.61–0.77)	0.69 (0.60–0.77)	0.31 (0.27–0.35)	0.69 (0.66–0.72)
		AdaBoost	0.67 (0.56–0.78)	0.20 (0.00–0.42)	0.81 (0.69–0.93)	0.35 (0.20–0.50)	0.51 (0.43–0.58)
		SVC	0.78 (0.61–0.96)	0.30 (0.08–0.52)	0.90 (0.84–0.96)	0.40 (0.27–0.54)	0.60 (0.50–0.69)
Girls	36/235	RF	0.53 (0.40–0.65)	0.72 (0.65–0.90)	0.32 (0.21–0.43)	0.25 (0.21–0.29)	0.52 (0.46–0.57)
		RF (OOB)	0.51 (0.49–0.53)	0.81 (0.72–0.89)	0.36 (0.27–0.45)	0.30 (0.30–0.31)	0.59 (0.57–0.60)
		AdaBoost	0.48 (0.35–0.61)	0.30 (0.14–0.46)	0.69 (0.59–0.79)	0.26 (0.20–0.31)	0.49 (0.43–0.55)
		SVC	0.51 (0.34–0.68)	0.17 (0.05–0.28)	0.83 (0.78–0.89)	0.29 (0.19–0.40)	0.50 (0.44–0.56)

*Note:* The values represent the validation data performance results obtained through 10‐fold cross‐validation. For the random forest (RF) model, performance metrics estimated using out‐of‐bag (OOB) observations are also included.

Abbreviations: AdaBoost, adaptive boosting; AUC, area the under receiver operating characteristic curve; SVC, support vector classifier.

**Figure 2:**
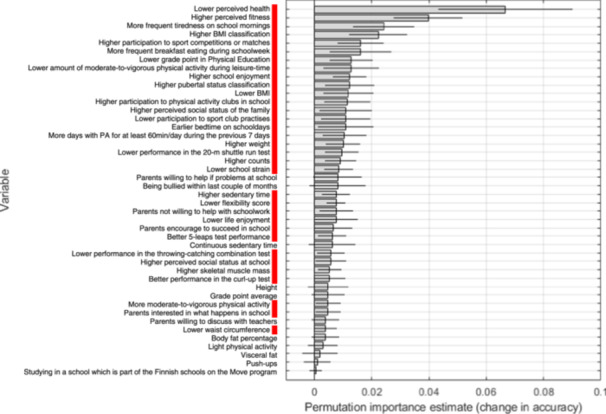
Permutation importance estimates for girls in the *selected set* for *all sites* (AUC 0.65). Red panel, risk factors; PA, physical activity; counts, accelerometer total activity counts.

The default tasks where prediction ability reached above random level (AUC 95% CI > 0.5) were further analyzed for variable importance. Altogether, 33 variables out of 48 baseline variables showed predictive power for whole body multisite pain incidence among girls. All variables are illustrated in Figure 2, and the top 10 are described in detail here. Poorer perceived health, higher perceived fitness, more frequent tiredness on schoolday mornings, having overweight or obesity based on body mass index, more frequent participation in sports competitions and matches, more frequent breakfast eating during the school week, a lower grade point in physical education, a lower amount moderate‐to‐vigorous physical activity during leisure time, higher school enjoyment, and higher pubertal status increased the probability of multisite pain incidence in the whole body area in girls.

Prediction ability with the Move! variables reached above the random level only in boys and in the whole body area (AUC 0.59 [0.56–0.62], Supporting Information S1: Table S1) and indicated that better muscular and cardiorespiratory fitness but poorer motor fitness predict higher multisite pain incidence (Supporting Information S1: Figure S1). With the full available data set, ML was able to predict multisite pain incidence only in girls (AUC 0.68 [0.66 to 0.70] in the whole body area (Supporting Information S1: Table S2). With the full data, along with physical, psychosocial, and lifestyle factors, individual pain sites at baseline rose as predictive factors of future multisite pain (Supporting Information S1: Figure S2). Balancing the data artificially with SMOTE‐NC did improve prediction ability up to AUC 0.69 (with high a standard deviation as a result of the small size of each validation fold), but due to automatic risk threshold selection designed for earlier tasks, it created in general suboptimal sensitivity and specificity values (Supporting Information S1: Tables S3–5).

### 3.2 Multisite Musculoskeletal Pain Incidence

Multisite pain incidence in the musculoskeletal area was 9.6% and 15.3% in boys and girls, respectively (Table 2). The prediction ability for multisite musculoskeletal pain incidence was AUC 0.65 (0.62–0.68) in boys and 0.51 (0.48–0.54) in girls (Table 2). Incorporating additional ML‐methods (AdaBoost, SVC) did not provide systematically better prediction ability across all performance metrics, although AUC reached 0.78 (0.61–0.96) with SVC in boys (Table 2).

**Figure 3:**
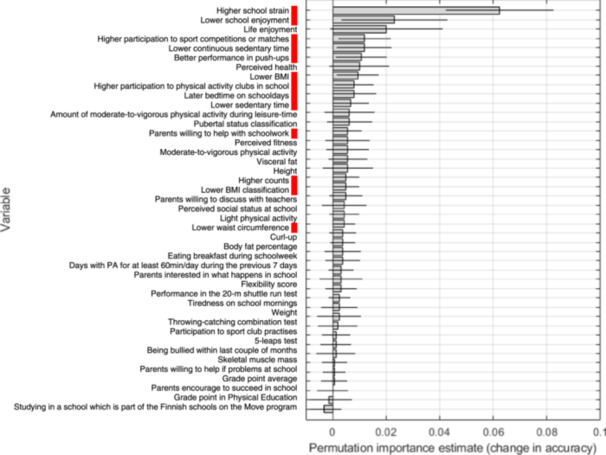
Permutation importance estimates for boys in the *selected set* for *musculoskeletal sites* (AUC 0.64). Red panel, risk factors; PA, physical activity; counts, accelerometer total activity counts.

In boys, a total of 13 variables out of 48 showed predictive power for multisite musculoskeletal pain incidence. The top 10 predictors for pain incidence included higher school strain, lower school enjoyment, a higher participation rate in sports competitions or matches, lower amounts of continuous device‐measured sedentary time, better muscular fitness measured with the number of push‐ups conducted within 1 min, a lower body mass index, more active participation in physical activity clubs in school, a later bedtime on schooldays, lower total sedentary time, and parents' higher willingness to help with schoolwork (Figure 3).

With the full available data set, ML was able to predict multisite musculoskeletal pain incidence only in girls (AUC 0.58 [0.56–0.60], Supporting Information S1: Table 2). With the full data, along with physical, psychosocial, and lifestyle factors, individual pain sites at baseline rose as predictive factors of future multisite pain (Supporting Information S1: Figure 3). Balancing the data artificially with SMOTE‐NC did also improve prediction ability of multisite musculoskeletal pain up to AUC 0.72, but showed in general similar suboptimal sensitivity and specificity values as for the whole body area (Supporting Information S1: Tables S3–5).

## 4 Discussion

In this data‐driven exploratory study, we aimed to investigate determinants of multisite pain incidence among the adolescent population with a novel ML approach. Multisite pain incidence in the study population was considerable, with up to 16% of boys and 28% of girls developing multisite weekly pain during the 2‐year follow‐up. The prediction ability of the ML approach with selected predictive variables reached with our default method an AUC 0.65 at its highest. With ML, a broad variety of variables predicting multisite pain incidence in adolescents were identified. Out of 48 selected variables, up to 33 variables showed predictive power in girls and 13 in boys. These findings highlight that rather than any isolated variable, a variety of factors may possess an increased risk for multisite pain and indicate the paradoxical nature of some variables, especially in girls.

Multisite pain is a major adverse health outcome in the adolescent population, affecting the daily lives of more than every fourth adolescent and their families [1]. Predicting the future onset of multisite pain, identifying individuals potentially experiencing disabling pain in the future, and recognizing the predictors of pain hold the potential to enhance the quality of life in this important demographic through better health education and policies. Pediatric experts have long stressed the importance of pain research and further understanding of pain epidemiology and underlying pathophysiology through innovative study designs [5].

ML‐based pattern recognition algorithms, a subgroup of artificial intelligence, have emerged as promising alternatives to traditional statistical methods in developing next‐generation tools to enhance public health. In contrast to theory‐based and often restricted traditional statistical models, the ML approach enables near unlimited learning capacity from the available data [27], providing the potential to develop more precise methods for screening and predicting adverse health outcomes. ML‐based approaches are acknowledged to hold significant potential for reforming public health policies in the future [32].

Previous studies have shown that prediction of multisite pain incidence is demanding [13], and the isolated correlates have modest effect sizes [8]. In this current study, the ML approach was able to predict pain incidence above the random level, however remaining with most methods under clinical relevance (AUC < 0.7) [33]. Through the ML approach, we found various predictors for multisite pain incidence, reflecting the previously reported physical, lifestyle, and psychosocial correlates [1, 2, 12, 13, 14, 15], and complementing these findings by illustrating the risky variables in a holistic framework alongside the paradoxical nature of some variables. For example, with whole body multisite pain incidence among girls, indicators of both lower and higher psychosocial wellbeing (e.g., lower life enjoyment vs. higher school enjoyment), low and high physical activity (lower amount of moderate‐to‐vigorous physical activity during leisure time vs. more days with physical activity for at least 60 min per day), lower body mass index, and obesity or overweight classification were identified as risk factors. These findings illustrate that risk factors, especially for whole body multisite pain incidence in girls are complex, associations are not linear, and individuals with both healthy and unhealthy lifestyles, favorable or unfavorable psychosocial status might develop multisite pain in the future. In boys, findings indicated more consistently that poorer psychosocial wellbeing, higher physical activity, a leaner body, and better physical fitness predict multisite musculoskeletal pain incidence and support the acknowledgment of overall wellbeing and health‐enhancing physical activity practices among boys to prevent musculoskeletal pain.

The strengths of this study were the novel application of ML in pain prediction, the longitudinal study design, and the extensiveness of predictors. The ML approach considerably extends pain research and provides potential avenues for screening and modeling complex phenomena in the future. The data was however limited by information (no data on current medication) and cases (e.g., < 66 cases in the data set) with possibly influencing the generalizability of the findings. ML explores patterns in the data and does not explain underlying mechanisms or causality. As an additional note, it is important to consider that the OOB values were utilized during the hyperparameter optimization process. This means that the model has already been exposed to these observations while tuning its parameters. Consequently, the performance estimates derived from the OOB values may be slightly biased, as the model is indirectly optimized to perform as well as possible on these observations. This potential bias should be considered when interpreting the results, as it may lead to an overestimation of the model's true generalization performance. However, this bias is probably very low, as Probst et al. [34] showed that optimizing the hyperparameters in an RF model gave, on average, only a 0.01 increase in the AUC metric. The multicollinearity of the variables might affect the interpretation of variables with similar phenomenal origins. Self‐reported data may suffer from recall bias, although the reliability of the utilized questionnaire has shown to be reasonable [22].

In conclusion, these novel findings highlight the multifaceted predictors of multisite pain incidence in adolescents and support the adoption of holistic and multidisciplinary prevention approaches in the future.

## Author Contributions

Laura Joensuu: conceptualization (equal), funding acquisition (supportive), investigation (supporting), project administration (lead), visualization (supporting), writing–original draft preparation (equal), review & editing (equal). Ilkka Rautiainen: conceptualization (equal), funding acquisition (supportive), data curation (lead), formal analysis (lead), methodology (lead), visualization (lead), writing–original draft preparation (equal), review & editing (equal). Arto J. Hautala: conceptualization (equal), supervision (equal), writing–original draft preparation (equal), review & editing (equal). Kirsti Siekkinen: conceptualization (equal), investigation (supporting), supervision (equal), writing–original draft preparation (equal), review & editing (equal). Katariina Pirnes: conceptualization (equal), supervision (equal), writing–original draft preparation (equal), review & editing (equal). Tuija H. Tammelin: conceptualization (equal), funding acquisition (lead), investigation (lead), project administration (supportive), writing–original draft preparation (equal), review & editing (equal).

## Conflicts of Interest

The authors declare no conflicts of interest.

## Transparency Statement

The lead author Laura Joensuu, Laura Joensuu affirms that this manuscript is an honest, accurate, and transparent account of the study being reported; that no important aspects of the study have been omitted; and that any discrepancies from the study as planned (and, if relevant, registered) have been explained.

## Supporting information

Supporting information.

## Data Availability

The data that support the findings of this study are available from the corresponding author upon reasonable request. Data and utilized scripts are available upon reasonable request from IR (scripts) and THT (data).
