# Supplementary material for: Prediction of Multisite Pain Incidence in Adolescence Using a Machine Learning Approach: A 2‐Year Longitudinal Study
Source: Health Sci Rep. 2024 Dec 10;7(12):e70252. doi: 10.1002/hsr2.70252 (PMC11628870; doi:10.1002/hsr2.70252)
Supplement: Supplementary file 1 — Supporting information. [file HSR2-7-e70252-s001.pdf]

Supplementary results and methods

Prediction of multisite pain incidence in adolescence using a machine learning approach: A 2-year longitudinal study

Laura Joensuu[a] Ph.D*, Ilkka Rautiainen[a] Ph.D.*, Arto J. Hautala[a] Ph.D., Kirsti Siekkinen[b] MSc, Katariina Pirnes[a] Ph.D, Tuija H Tammelin[b] Ph.D.

[a]Faculty of Sport and Health Sciences, University of Jyväskylä, Jyväskylä, Finland, [b]Likes, Jamk University of Applied Sciences, Jyväskylä, Finland

*Authors contributed equally

3 eFigures

eFigure 1. Permutation importance estimates for boys in the *Move! set* for *all sites* (AUC 0.59).
eFigure 2. Permutation importance estimates for girls in the *full set* for *all sites* (AUC 0.68).
eFigure 3. Permutation importance estimates for girls in the *full set* for *musculoskeletal sites* (AUC 0.58).
eFigure 4. Average absolute SHAP values for girls in the main article set for all sites (AdaBoost) (AUC 0.60).
eFigure 5. Swarm chart of SHAP values for girls in the main article set for all sites (AdaBoost) (AUC 0.60).
eFigure 6. Average absolute SHAP values for girls in the main article set for all sites (SVC) (AUC 0.63).
eFigure 7. Swarm chart of SHAP values for girls in the main article set for all sites (SVC) (AUC 0.63).
eFigure 8. Average absolute SHAP values for boys in the main article set for musculoskeletal sites (AdaBoost) (AUC 0.67)
eFigure 9. Swarm chart of SHAP values for boys in the main article set for musculoskeletal sites (AdaBoost) (AUC 0.67)
eFigure 10. Average absolute SHAP values for boys in the main article set for musculoskeletal sites (SVC) (AUC 0.78)
eFigure 11. Swarm chart of SHAP values for boys in the main article set for musculoskeletal sites (SVC) (AUC 0.78)

5 eTables

eTable 1. Prediction ability of machine learning for multisite pain incidence among adolescents. Results for the Move! set.
eTable 2.Prediction ability of machine learning for multisite pain incidence among adolescents. Results for the full set.
eTable 3. Prediction ability of machine learning for multisite pain incidence among adolescents. Results for the Move! set, balanced with SMOTE-NC.
eTable 4. Prediction ability of machine learning for multisite pain incidence among adolescents. Results for the selected data set, balanced with SMOTE-NC.
eTable 5. Prediction ability of machine learning for multisite pain incidence among adolescents. Results for the full set, balanced with SMOTE-NC.

eTable 1. Prediction ability of machine learning for multisite pain incidence among adolescents. Results for the Move! set.

		Prediction ability		
**Multisite pain incidence**	**Cases/N**	**AUC (95 % CI)**	**Sensitivity**	**Specificity**
**All body sites**				
Boys	25/168	0.59 (0.56 to 0.62)	0.69 (0.60 to 0.78)	0.59 (0.50 to 0.68)
Girls	63/225	0.38 (0.34 to 0.41)	0.98 (0.98 to 0.98)	0.01 (0.00 to 0.02)
**Musculoskeletal sites**				
Boys	17/170	0.44 (0.40 to 0.47)	0.71 (0.53 to 0.90)	0.40 (0.18 to 0.63)
Girls	34/225	0.35 (0.31 to 0.40)	0.85 (0.72 to 0.99)	0.17 (0.00 to 0.34)

AUC results are estimated from the out-of-bag observations using the 10-fold cross-validation.

eTable 2. Prediction ability of machine learning for multisite pain incidence among adolescents. Results for the full set.

		Prediction ability		
**Multisite pain incidence**	**Cases/N**	**AUC (95 % CI)**	**Sensitivity**	**Specificity**
**All body sites**				
Boys	28/169	0.46 (0.42 to 0.50)	0.89 (0.85 to 0.93)	0.19 (0.13 to 0.26)
Girls	65/230	0.68 (0.66 to 0.70)	0.64 (0.55 to 0.74)	0.65 (0.52 to 0.78)
**Musculoskeletal sites**				
Boys	18/172	0.54 (0.49 to 0.59)	0.69 (0.56 to 0.82)	0.51 (0.37 to 0.65)
Girls	35/230	0.58 (0.56 to 0.60)	0.63 (0.53 to 0.73)	0.61 (0.49 to 0.73)

AUC results are estimated from the out-of-bag observations using the 10-fold cross-validation.

eTable 3. Prediction ability of machine learning for multisite pain incidence among adolescents. Results for the Move! set, balanced with SMOTE-NC.

		Prediction ability		
**Multisite pain incidence**	**Cases/N**	**AUC (95 % CI)**	**Sensitivity**	**Specificity**
**All body sites**				
Boys	25/168	0.65 (0.54 to 0.77)	0.35 (0.19 to 0.52)	0.80 (0.72 to 0.88)
Girls	63/225	0.44 (0.39 to 0.49)	0.39 (0.27 to 0.51)	0.54 (0.46 to 0.63)
**Musculoskeletal sites**				
Boys	17/170	0.52 (0.37 to 0.66)	0.15 (0.00 to 0.30)	0.85 (0.80 to 0.91)
Girls	34/225	0.30 (0.21 to 0.40)	0.17 (0.05 to 0.28)	0.64 (0.54 to 0.75)

Results are estimated from the validation set observations using the 10-fold cross-validation.

eTable 4. Prediction ability of machine learning for multisite pain incidence among adolescents. Results for the selected data set, balanced with SMOTE-NC.

		Prediction ability		
**Multisite pain incidence**	**Cases/N**	**AUC (95 % CI)**	**Sensitivity**	**Specificity**
**All body sites**				
Boys	28/175	0.61 (0.52 to 0.71)	0.00[a]	0.98 (0.95 to 1.00)
Girls	66/235	0.44 (0.39 to 0.49)	0.39 (0.27 to 0.51)	0.54 (0.46 to 0.63)
**Musculoskeletal sites**				
Boys	17/178	0.72 (0.61 to 0.82)	0.65 (0.40 to 0.91)	0.63 (0.50 to 0.76)
Girls	36/235	0.50 (0.42 to 0.58)	0.03 (0.00 to 0.10)	0.95 (0.92 to 0.98)

Results are estimated from the validation set observations using the 10-fold cross-validation. [a]Due to automated threshold selection that maximized the f-measure during training for OOB observations, some sensitivity and specificity values were taken from a suboptimal point in the ROC curve, leading to sensitivity values very close to or even equal to zero.

eTable 5. Prediction ability of machine learning for multisite pain incidence among adolescents. Results for the full set, balanced with SMOTE-NC.

		Prediction ability		
**Multisite pain incidence**	**Cases/N**	**AUC (95 % CI)**	**Sensitivity**	**Specificity**
**All body sites**				
Boys	28/169	0.61 (0.49 to 0.72)	0.07 (0.00 to 0.15)	0.97 (0.94 to 1.00)
Girls	65/230	0.69 (0.61 to 0.77)	0.09 (0.01 to 0.17)	0.96 (0.91 to 1.00)
**Musculoskeletal sites**				
Boys	18/172	0.60 (0.47 to 0.73)	0.00[a]	1.00[a]
Girls	35/230	0.54 (0.45 to 0.63)	0.03 (0.00 to 0.07)	0.98 (0.96 to 1.00)

Results are estimated from the validation set observations using the 10-fold cross-validation. [a]Due to automated threshold selection that maximized the f-measure during training for OOB observations, some sensitivity and specificity values were taken from a suboptimal point in the ROC curve, leading to sensitivity values very close to or even equal to zero.

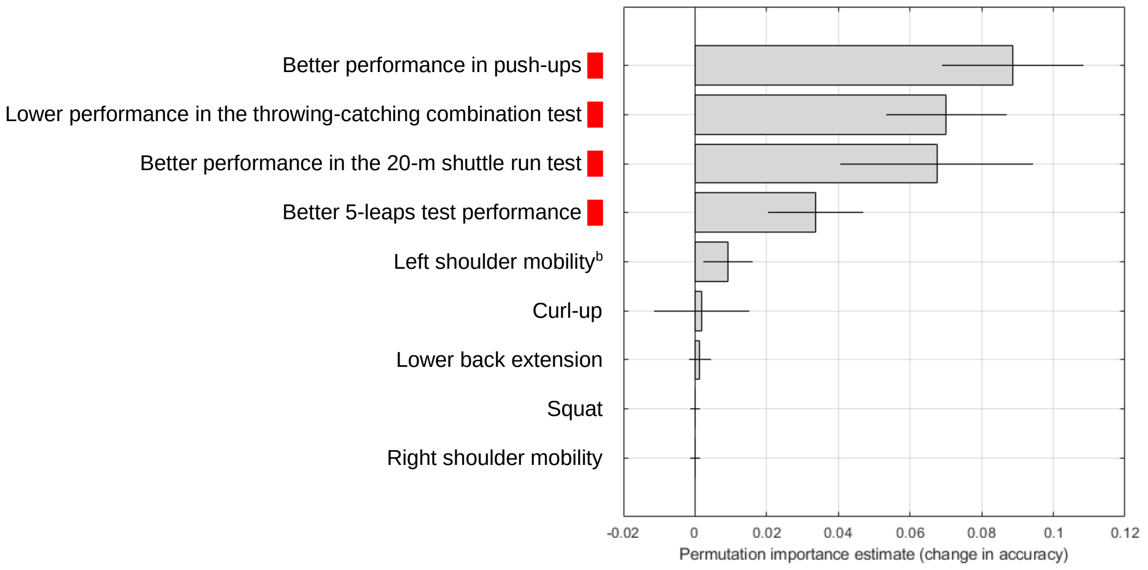

eFigure 1. Permutation importance estimates for boys in the *Move! set* for *all sites* (AUC 0.59). Red panel, risk factors; [b]Direction of the association not calculated for nominal variables

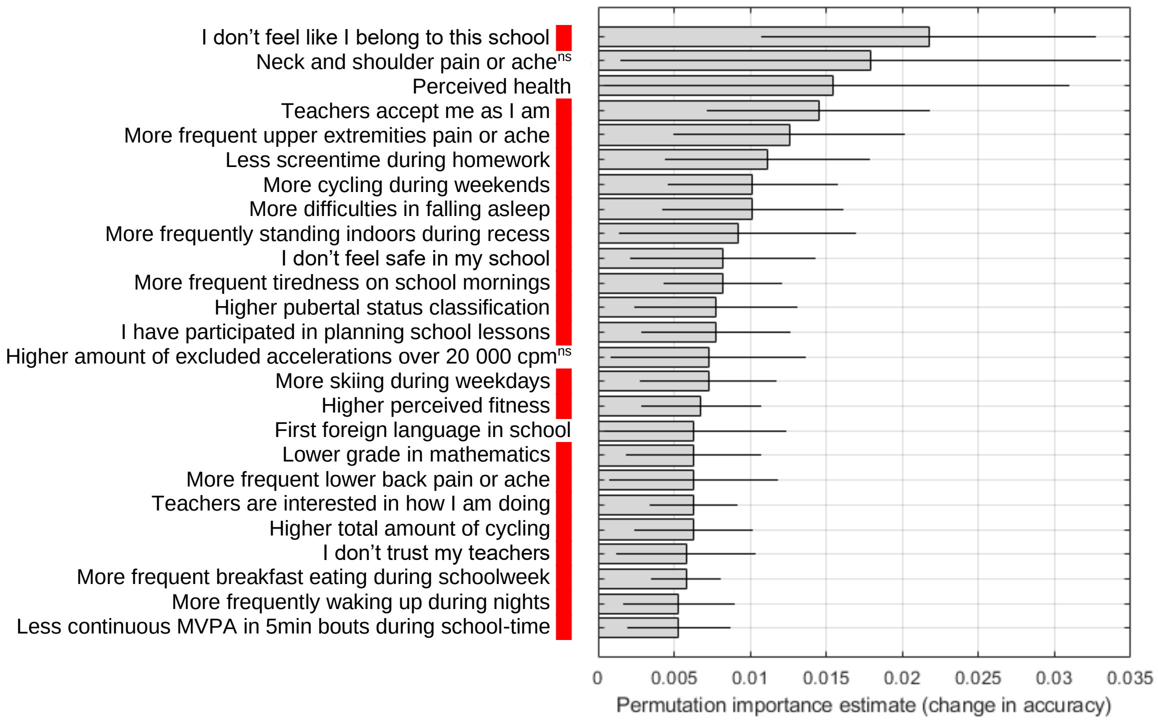

eFigure 2. Permutation importance estimates for girls in the *full set* for *all sites* (AUC 0.68). Only the top 25 predictors are presented. Red panel, risk factors; [ns]Not significant, variable significance calculated based on t-test in MATLAB, slightly differing from the manually calculated confidence intervals.

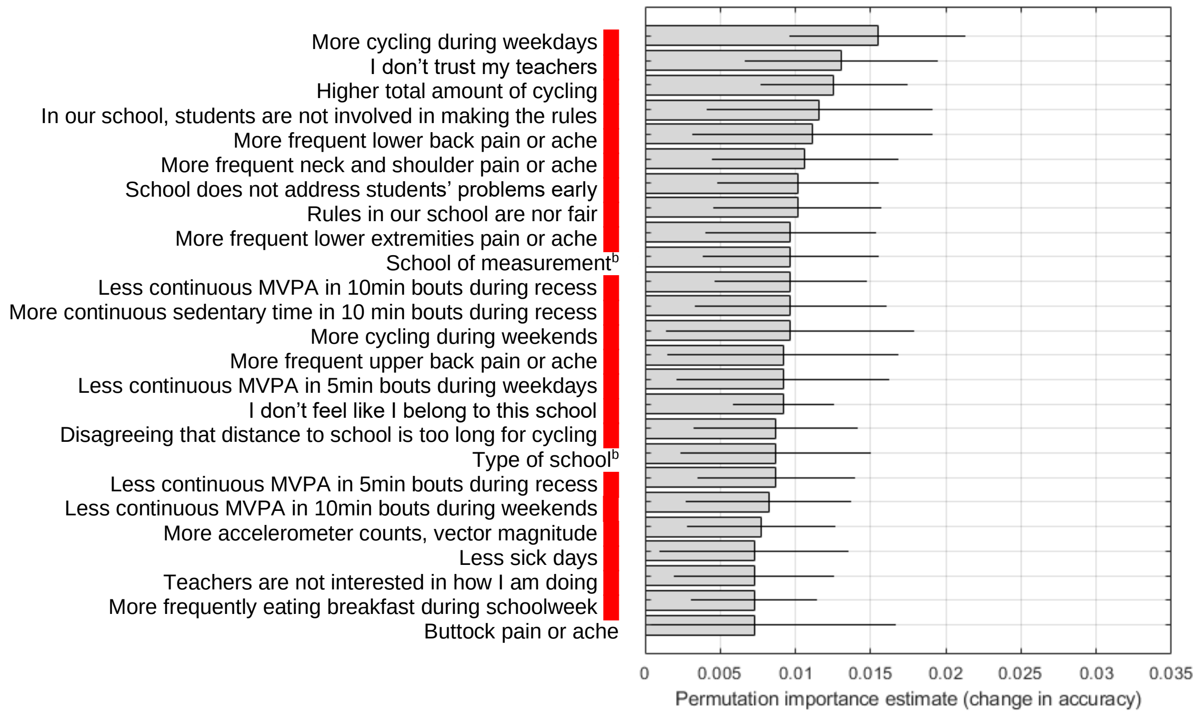

eFigure 3. Permutation importance estimates for girls in the *full set* for *musculoskeletal sites* (AUC 0.58). Only the top 25 predictors are presented. Red panel, risk factors; [b]Direction of the association not calculated for nominal variables

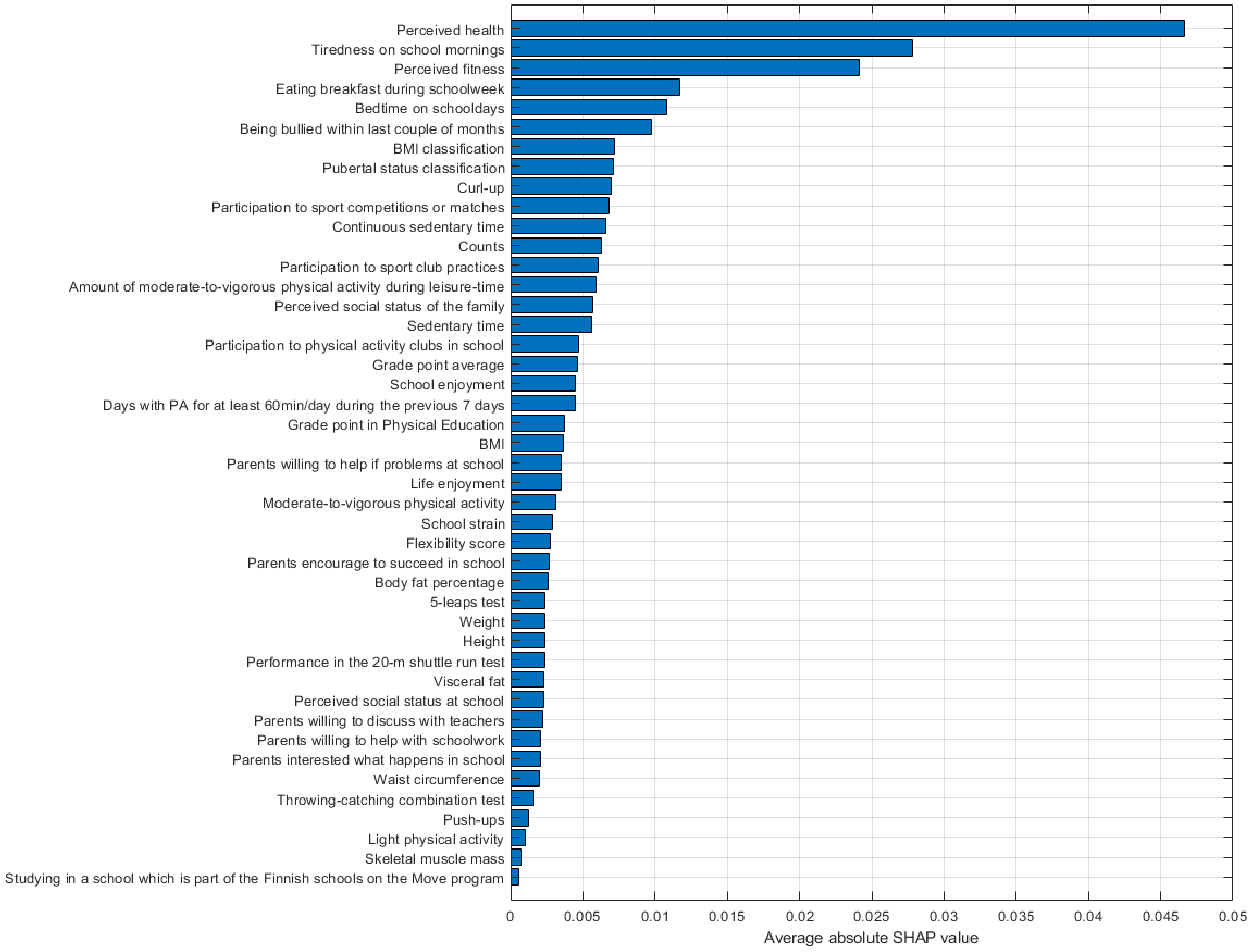

eFigure 4. Average absolute SHAP values for girls in the *main article set* for *all sites* (AdaBoost) (AUC 0.60). These values were derived by aggregating observations from the training data across the ten cross-validation folds.

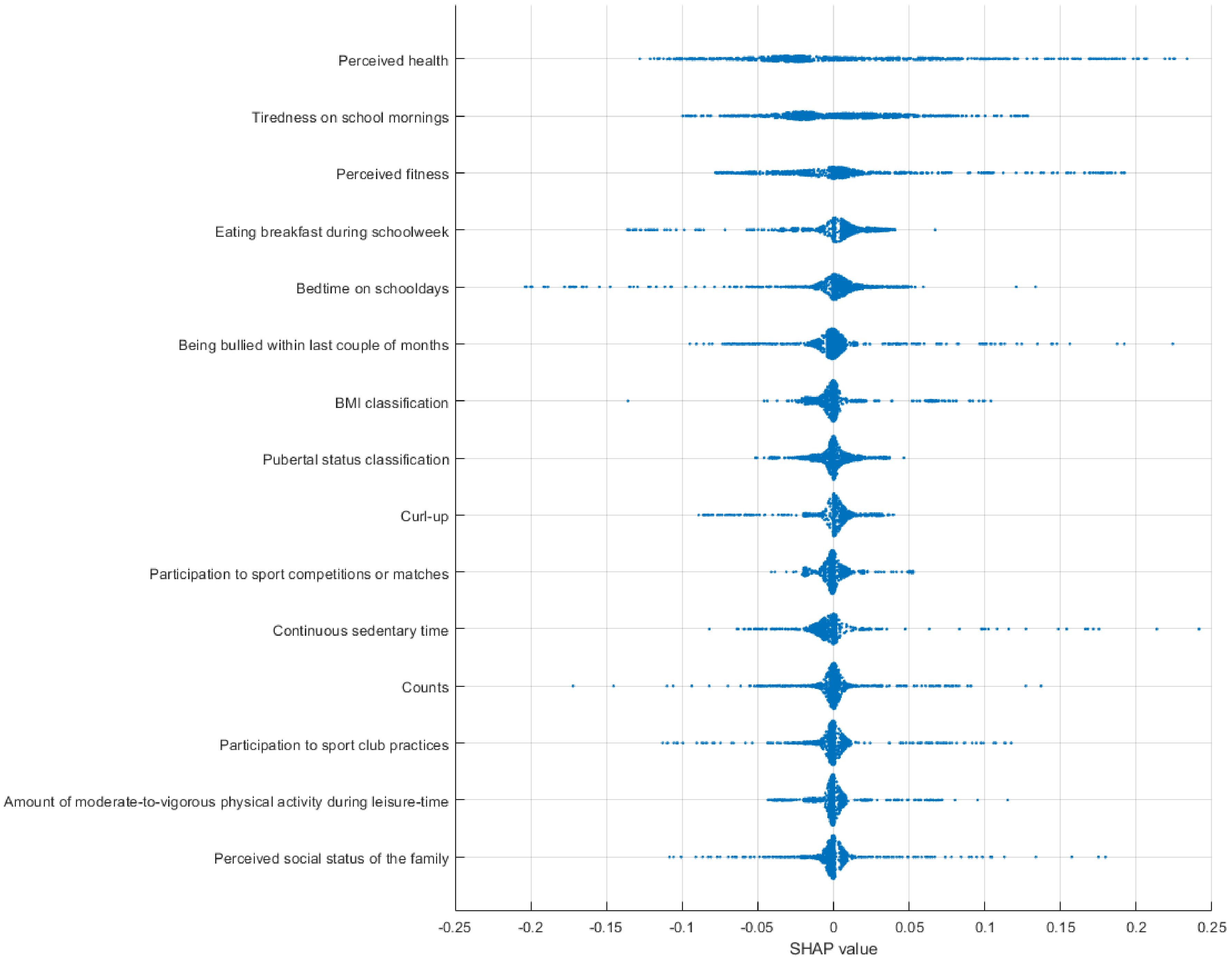

eFigure 5. Swarm chart of SHAP values for girls in the *main article set* for *all sites* (AdaBoost) (AUC 0.60). It shows the impact of each variable on the model's output. Each dot represents a single observation. These values were derived by aggregating observations from the training data across the ten cross-validation folds. The chart displays the top 15 variables, selected based on their absolute mean SHAP values.

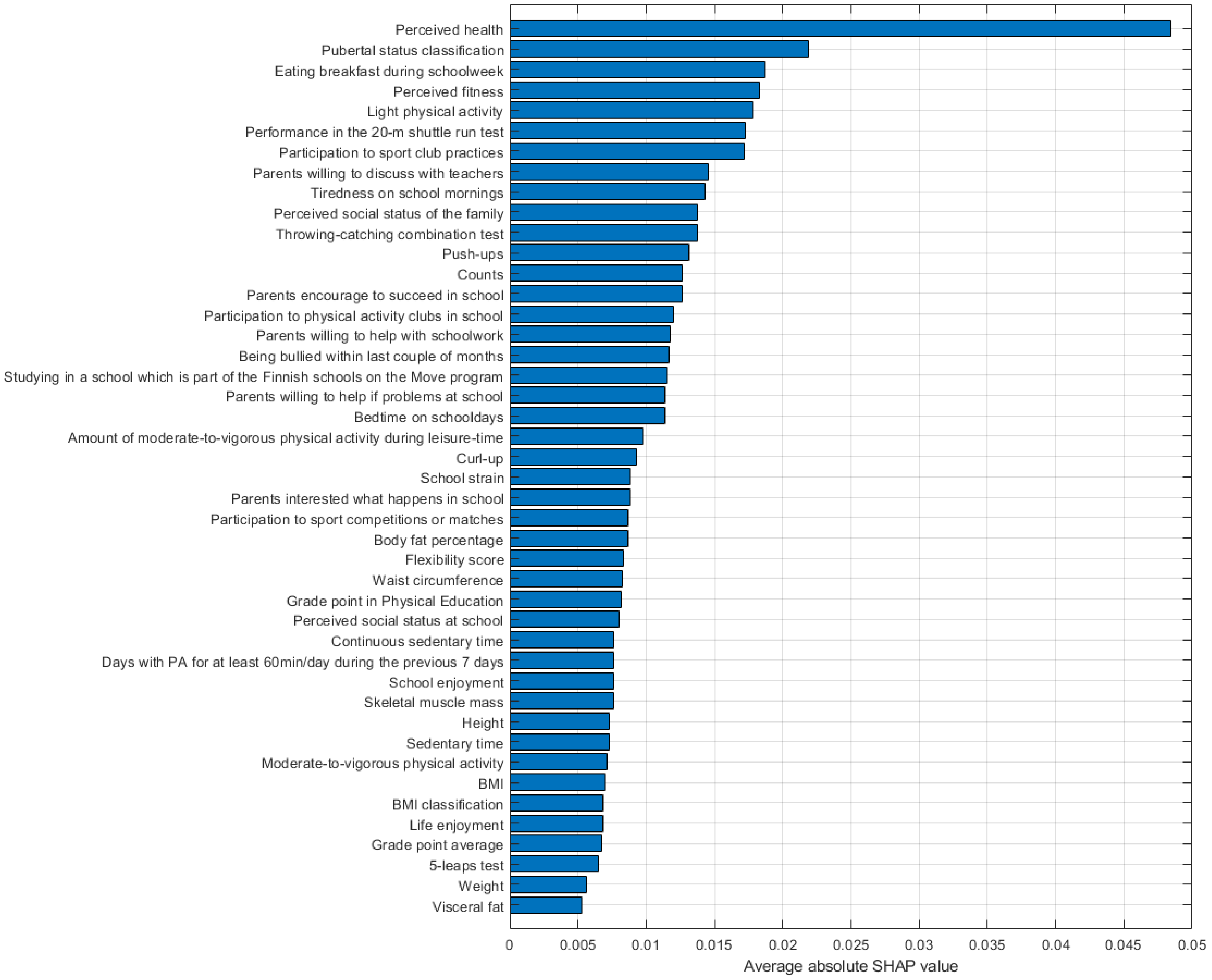

eFigure 6. Average absolute SHAP values for girls in the *main article set* for *all sites* (SVC) (AUC 0.63). These values were derived by aggregating observations from the training data across the ten cross-validation folds.

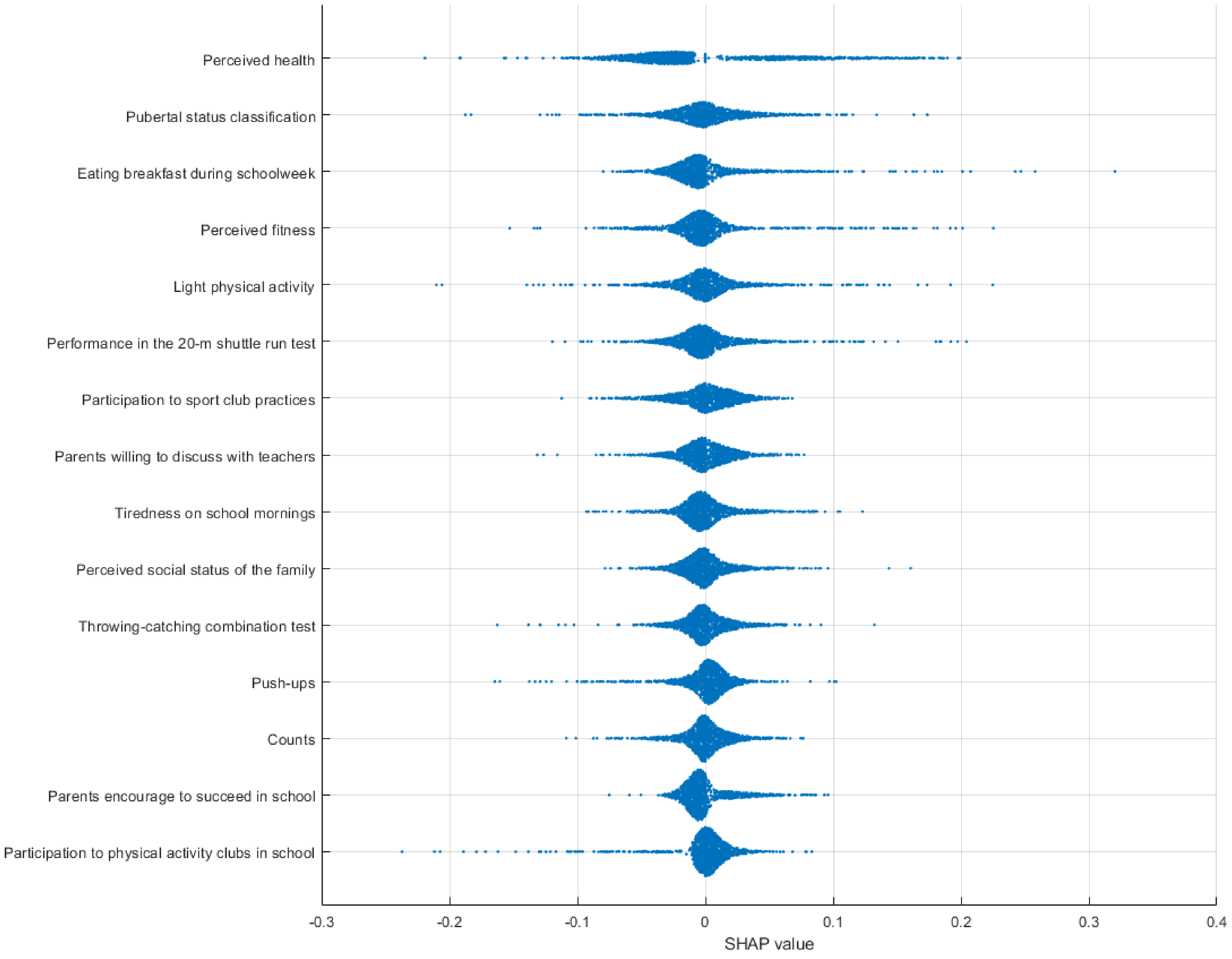

eFigure 7. Swarm chart of SHAP values for girls in the *main article set* for *all sites* (SVC) (AUC 0.63). The chart displays the top 15 variables, selected based on their absolute mean SHAP values. It shows the impact of each variable on the model's output. Each dot represents a single observation. These values were derived by aggregating observations from the training data across the ten cross-validation folds. The chart displays the top 15 variables, selected based on their absolute mean SHAP values.

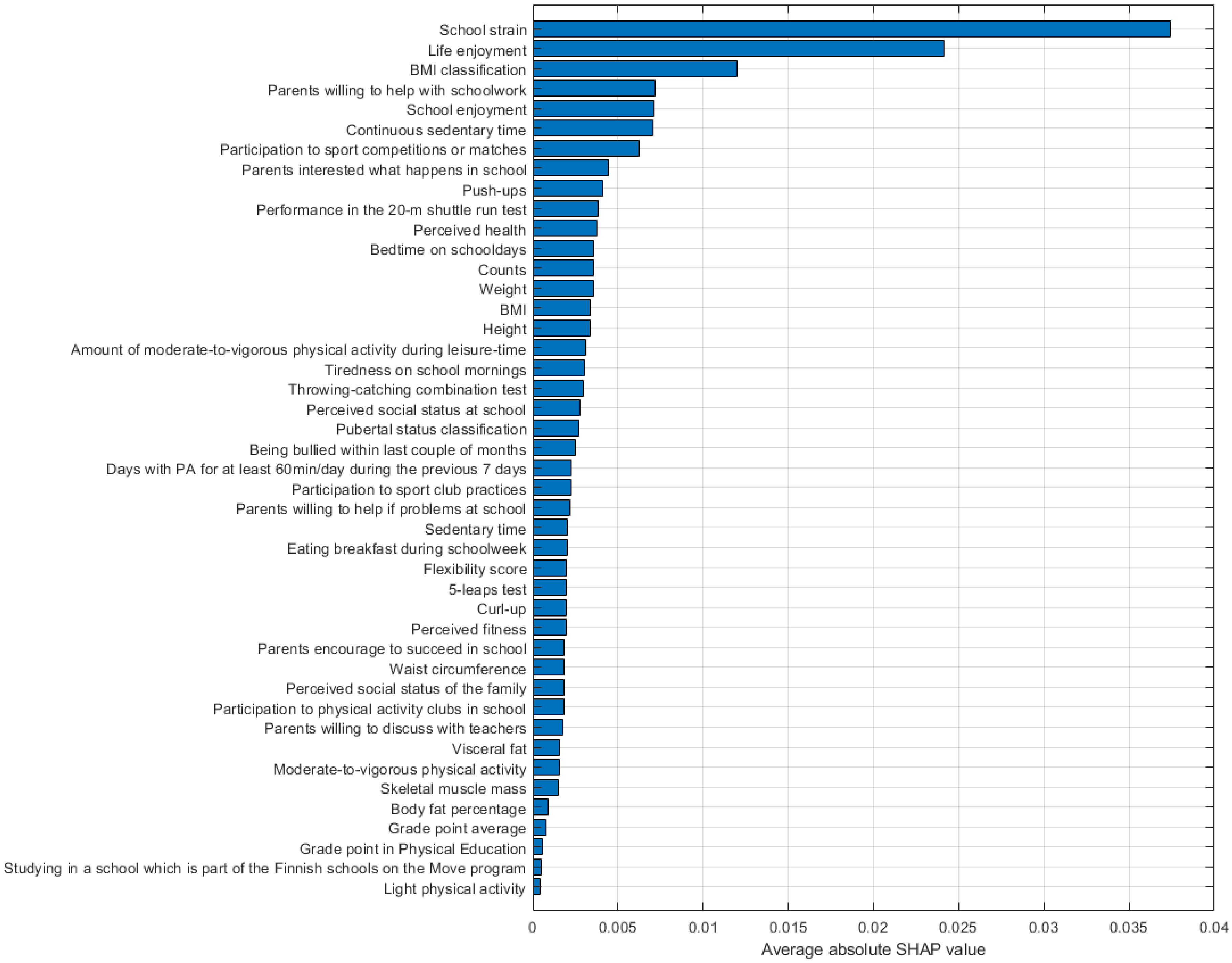

eFigure 8. Average absolute SHAP values for boys in the main article set for musculoskeletal sites (AdaBoost) (AUC 0.67). These values were derived by aggregating observations from the training data across the ten cross-validation folds.

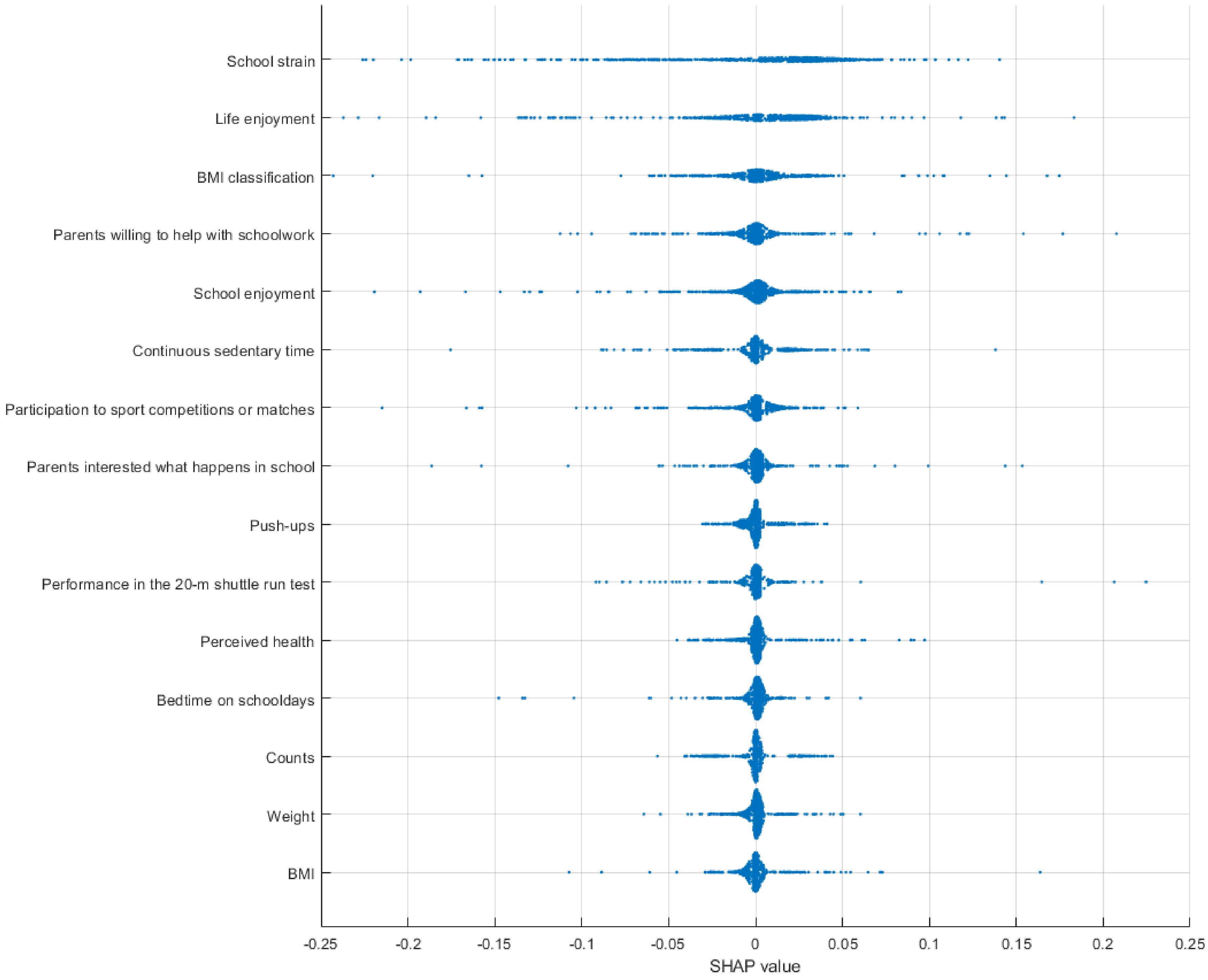

eFigure 9. Swarm chart of SHAP values for boys in the main article set for musculoskeletal sites (AdaBoost) (AUC 0.67). The chart displays the top 15 variables, selected based on their absolute mean SHAP values. It shows the impact of each variable on the model's output. Each dot represents a single observation. These values were derived by aggregating observations from the training data across the ten cross-validation folds. The chart displays the top 15 variables, selected based on their absolute mean SHAP values.

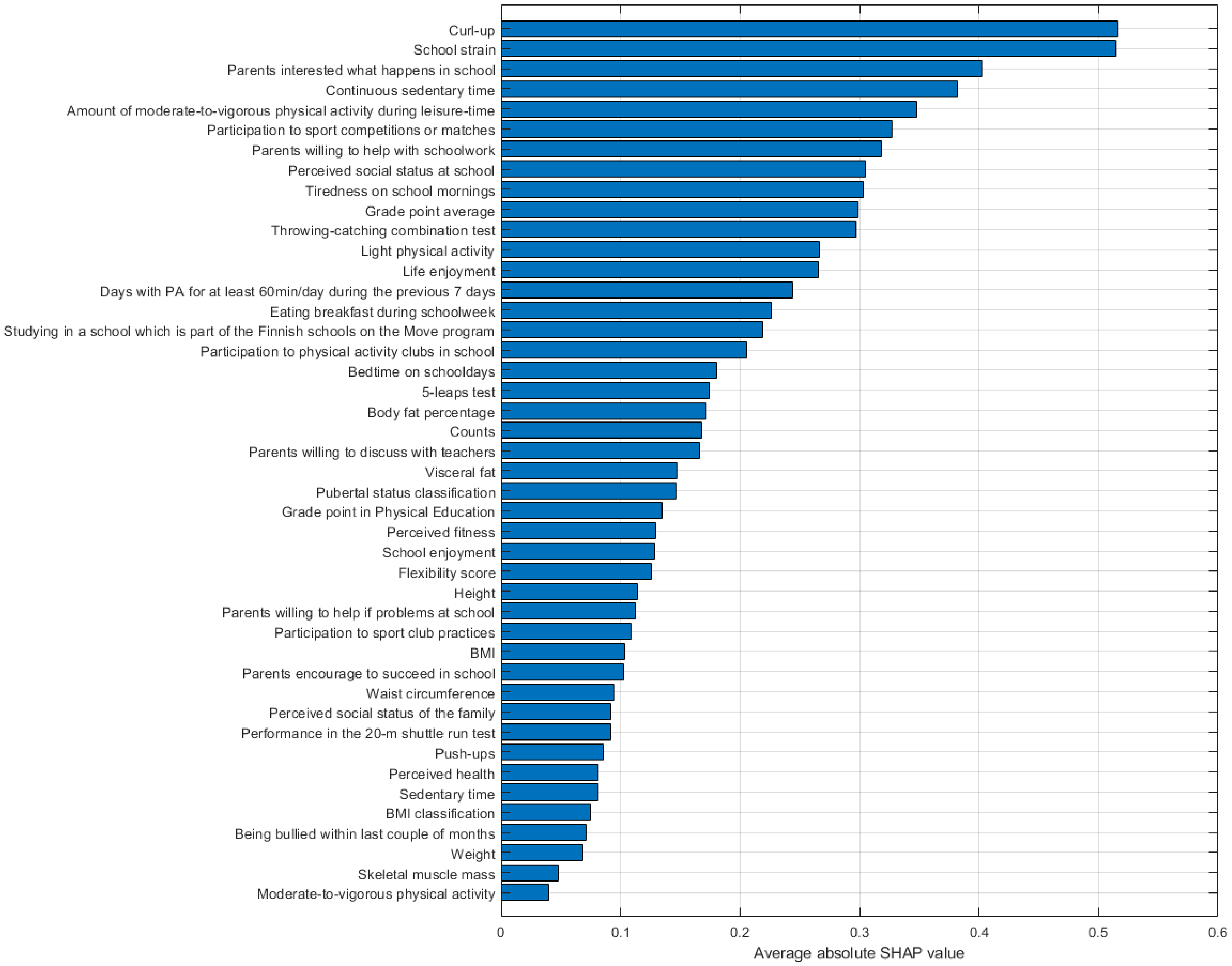

eFigure 10. Average absolute SHAP values for boys in the main article set for musculoskeletal sites (SVC) (AUC 0.78). These values were derived by aggregating observations from the training data across the ten cross-validation folds.

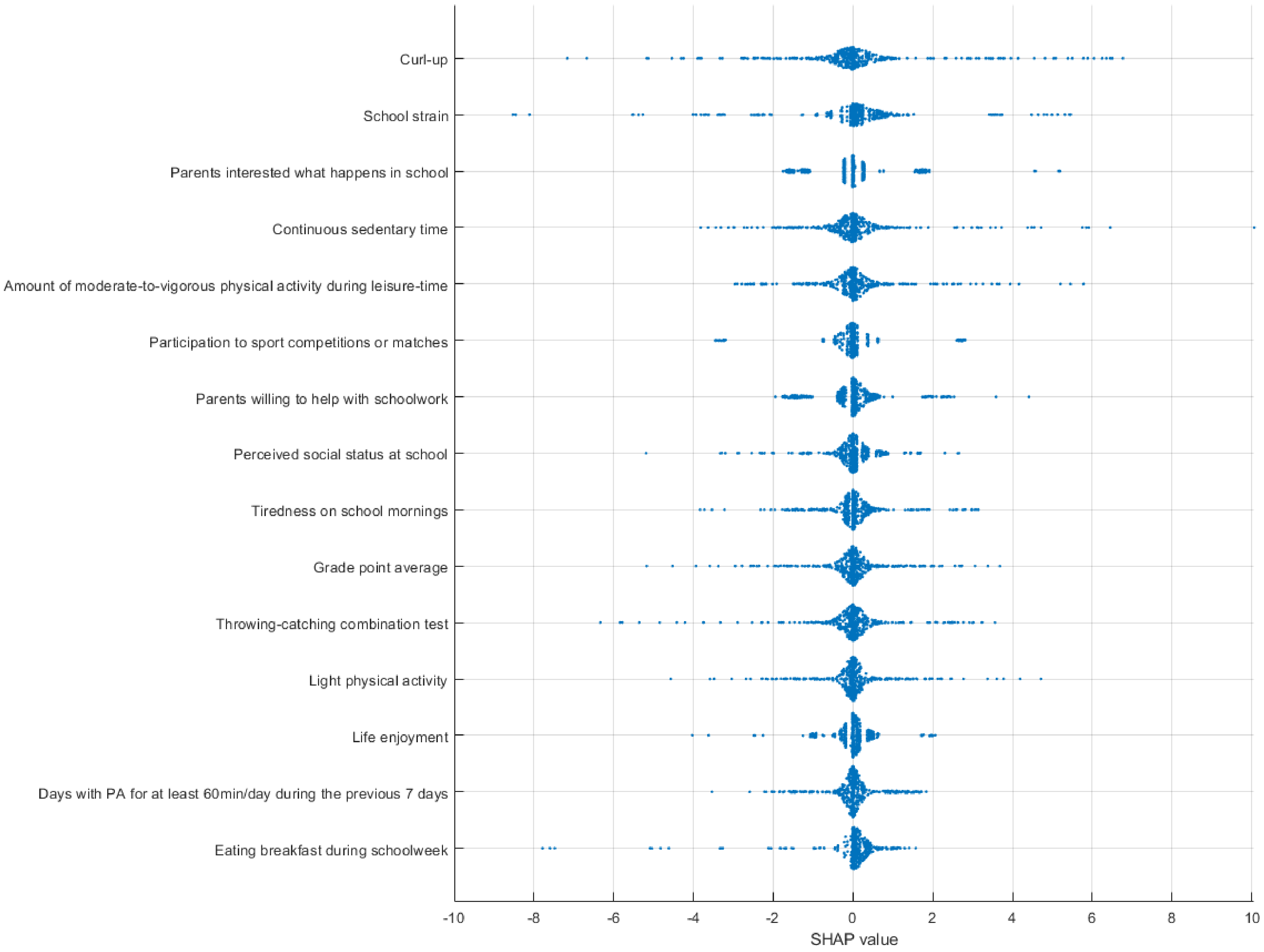

eFigure 11. Swarm chart of SHAP values for boys in the main article set for musculoskeletal sites (SVC) (AUC 0.78). The chart displays the top 15 variables, selected based on their absolute mean SHAP values. It shows the impact of each variable on the model's output. Each dot represents a single observation. These values were derived by aggregating observations from the training data across the ten cross-validation folds. The chart displays the top 15 variables, selected based on their absolute mean SHAP values.

Supplementary methods

The training phase included procedures to optimize *pain* class prediction with RF and the training data. Bayesian optimization was employed to estimate the optimal hyperparameters (e.g. the method parameters that must be defined beforehand) for RF. The F-measure, which balances the precision and sensitivity of the classifier by computing their harmonic mean, was used as the optimization target for the RF out-of-bag samples. It is defined as

$$F = 2 \times \frac{precision \times sensitivity}{precision + sensitivity},$$

where

$$precision = \frac{TP}{TP + FP}$$

and

$$sensitivity = \frac{TP}{TP+FN}.$$

In the above equations, TP (true positives) refers to the number of correctly detected *pain* class cases. FP (false positives) is the number of cases incorrectly identified as belonging to the *pain* class and FN (false negatives) is the number of cases incorrectly identified as belonging to the *no pain* class. Since the Bayesian optimization aims to minimize the given objective, the final target for optimization was $1 - F$.

The predicted probabilities of the RF model for the two classes were used to make receiver operating characteristic (ROC) curves, using OOB observations. During Bayesian optimization, the F-measure was taken from the point in the ROC curve that maximized the value. Afterwards, the best estimated hyperparameters were used when the RF model was trained again. The presented prediction results were then recorded using the OOB observations. Additionally, the threshold that maximized F-measure was recorded and later used when validating the results using the separate validation data portion in each fold. These separate results are presented only for the setup using SMOTE-NC, since the OOB estimates in RF are considered to be good for assessing prediction performance and generalizability.[1]

In addition to abovementioned equations, specificity, defined as

$$specificity = \frac{TN}{TN+FP},$$

was employed as a performance metric
After training, a validation phase was implemented where the validity of the findings was tested against the left-out fold in 10-fold CV. During this phase, the measures used to estimate the prediction performance were AUC, sensitivity and specificity.

Additionally, accuracy, defined as

$$accuracy = \frac{TP + TN}{TP + FP + TN + FN}$$

was utilized in estimating the variable importances. Mean change in accuracy was used as the estimate for individual variable importance. Another performance metric used was balanced accuracy, which adjusts the accuracy measure to account for imbalanced data. For a two-class problem, it is defined as

$$\text{balanced accuracy} = \frac{1}{2}\left(\frac{TP}{TP + FN} + \frac{TN}{TN + FP}\right).$$

For handling the missing values in data, the original random forest method suggested two ways of imputing the missing values.[1] The TreeBagger implementation in MATLAB employs a surrogate decision split especially for handling the missing values in data. When the surrogate decision splits flag is set to “on”, a similar or correlated predictor value is used instead of the missing value.

As an additional note, it is important to consider that the OOB values were utilized during the hyperparameter optimization process. This means that the model has already been exposed to these observations while tuning its parameters. Consequently, the performance estimates derived from the OOB values may be slightly biased, as the model is indirectly optimized to perform well on these observations. This potential bias should be considered when interpreting the results for the *full data set* and *Move! variable set*, as it may lead to an overestimation of the model’s true generalization performance.

Random forest (RF) hyperparameters

Four RF hyperparameters in MATLAB's TreeBagger function were optimized:
1. *NumPredictorsToSample*: The number of variables to select at random for each decision split (range to search was from 1 to *total_number_of_variables_in_data-1*)
2. *MinLeafSize*: The minimum number of observations per tree leaf (range from 2 to 15).
3. *MaxNumSplits*: The maximum number of decision splits (range from 1 to 30).
4. *Surrogate*: Surrogate decision splits flag (options included on, off and all).

Static modified RF parameters included:
1. The number of trees in the forest was set to 500.
2. Nominal variables in the data were set as categorical variables (option *CategoricalPredictors*).
3. Algorithm used to select the best split predictor (option *PredictorSelection*) was set to *interaction-curvature*.

In addition, two static parameters were modified in MATLAB's Bayesian optimization (bayesopt) function:
1. *MaxObjectiveEvaluations* was set to 100 (*main article set*) or 30 (*Move! variable set* and *full data set*), meaning that there are 100 or 30 iterations to search for optimal hyperparameters, after which the optimization was terminated.
2. *AcquisitionFunctionName* was set to expected-improvement-plus.

AdaBoost hyperparameters

Within each 10-fold cross-validation fold, optimal hyperparameters for AdaBoost were determined by further dividing the training data (90%) into five folds (nested 5-fold CV, with 80% as training data and 20% as validation data). The 5-fold average F-measure on the nested validation data was used to estimate the model's performance for the current hyperparameter setup.

AdaBoost constructs the model using multiple weak learners, with decision trees serving as the weak learners in our models. Bayesian hyperparameter optimization was employed to search for the following four hyperparameters:

1. *MaxNumSplits*: The maximum number of decision splits in the decision tree weak learner (range from 1 to 30).
2. *Surrogate*: The surrogate decision splits flag in the decision tree weak learner (options included on, off and all).
3. *LearnRate*: The learning rate shrinkage (logarithmic range from 0.001 to 1).
4. *NumLearningCycles*: The number of weak learners in the model (range from 10 to 500).

MaxObjectiveEvaluations was set to 100.

Support vector classifier (SVC) hyperparameters

Similarly to AdaBoost, within each 10-fold cross-validation fold, optimal hyperparameters for SVC were determined by further dividing the training data (90%) into five folds (nested 5-fold CV, with 80% as training data and 20% as validation data). The 5-fold average F-measure on the nested validation data was used to estimate the model's performance for the current hyperparameter setup.

Bayesian hyperparameter optimization was employed to search for the following four hyperparameters:

1. *BoxConstraint*: Regularization parameter (logarithmic range from 0.001 to 1,000).
2. *KernelFunction*: Gaussian or linear kernel.
3. *KernelScale*: A scaling factor used to normalize the data before evaluating the kernel function (logarithmic range from 0.001 to 1,000).
4. *Standardize*: Whether to standardize the data or not (true or false).

MaxObjectiveEvaluations was set to 100.

References

1. Breiman L. Random Forests. *Mach Learn*. 2001;45(1):5-32. doi:10.1023/A:1010933404324
